# Interplay between nitric oxide and gonadotrophin-releasing hormone in the neuromodulation of the corpus luteum during late pregnancy in the rat

**DOI:** 10.1186/s12958-022-00894-6

**Published:** 2022-01-26

**Authors:** Sandra Vallcaneras, Laura Morales, María Belén Delsouc, Darío Ramirez, Verónica Filippa, Marina Fernández, Carlos M. Telleria, Marilina Casais

**Affiliations:** 1 Laboratorio de Biología de la Reproducción (LABIR), Facultad de Química, Bioquímica y Farmacia, Universidad Nacional de San Luis, Instituto Multidisciplinario de Investigaciones Biológicas de San Luis (IMIBIO-SL-CONICET), Av. Ejército de los Andes 950, CP D5700HHW San Luis, Argentina; 2 Laboratorio de Medicina Experimental & Traduccional (LME&T), Facultad de Química, Bioquímica y Farmacia, Universidad Nacional de San Luis, Instituto Multidisciplinario de Investigaciones Biológicas de San Luis (IMIBIO-SL-CONICET), Av. Ejército de los Andes 950, CP D5700HHW San Luis, Argentina; 3 Laboratorio de Histología, Facultad de Química, Bioquímica y Farmacia, Universidad Nacional de San Luis, Ejercito de los Andes 950, Bloque I, Piso No. 1, 5700 San Luis, Argentina; 4 Consejo Nacional de Investigaciones Científicas y Técnicas (CONICET), 5700 San Luis, Argentina; 5 Instituto de Biología y Medicina Experimental (IBYME-CONICET), V. de Obligado 2490, C1428ADN Buenos Aires, Argentina; 6 Experimental Pathology Unit, Department of Pathology, Faculty of Medicine and Health Sciences, McGill University, 3775 University Street, Duff Medical Sciences Building, Laboratory B22, Montreal, Quebec PC H3A 2B4 Canada

**Keywords:** Nitric oxide, Gonadotrophin-releasing hormone, Ex-vivo coeliac ganglion-superior ovarian nerve-ovary system, End of pregnancy, Corpus luteum, Rat

## Abstract

**Background:**

Nitric oxide and GnRH are biological factors that participate in the regulation of reproductive functions. To our knowledge, there are no studies that link NO and GnRH in the sympathetic ganglia. Thus, the aim of the present work was to investigate the influence of NO on GnRH release from the coeliac ganglion and its effect on luteal regression at the end of pregnancy in the rat.

**Methods:**

The ex vivo system composed by the coeliac ganglion, the superior ovarian nerve, and the ovary of rats on day 21 of pregnancy was incubated for 180 min with the addition, into the ganglionic compartment, of L-N^G^-nitro arginine methyl ester (L-NAME), a non-selective NO synthase inhibitor. The control group consisted in untreated organ systems.

**Results:**

The addition of L-NAME in the coeliac ganglion compartment decreased NO as well as GnRH release from the coeliac ganglion. In the ovarian compartment, and with respect to the control group, we observed a reduced release of GnRH, NO, and noradrenaline, but an increased production of progesterone, estradiol, and expression of their limiting biosynthetic enzymes, 3β-HSD and P450 aromatase, respectively. The inhibition of NO production by L-NAME in the coeliac ganglion compartment also reduced luteal apoptosis, lipid peroxidation, and nitrotyrosine, whereas it increased the total antioxidant capacity within the corpora lutea.

**Conclusion:**

Collectively, the results indicate that NO production by the coeliac ganglion modulates the physiology of the ovary and luteal regression during late pregnancy in rats.

**Supplementary Information:**

The online version contains supplementary material available at 10.1186/s12958-022-00894-6.

## Background

In the ovary, the corpus luteum (CL) is a transitory glandular structure, which is essential for the maintenance of pregnancy, given its ability to secrete progesterone. The function and lifespan of this dynamic gland depends on the balance between luteotrophic and luteolytic factors, such as steroid and peptide hormones, growth factors, gonadotropins, prostaglandins, cytokines, neuropeptides, and reactive oxygen species (ROS), among others [1]. At the end of pregnancy in the rat, the CL undergoes a regression process characterized by decreased functionality and structural involution [2]. Given the importance of this process for the homeostasis of the ovarian tissue, and to ensure the successful delivery of the fetuses, a series of autocrine and paracrine signals participate in its regulation.

Nitric oxide (NO) is a key cellular signaling factor in reproductive physiology. Several studies have demonstrated that NO participates in the control of gonadotrophin-releasing hormone (GnRH) production modulating the hypothalamo-pituitary-ovary axis. Chachlaki et al. [3] showed that the administration in vitro of a NO donor to hypothalamic explants stimulated GnRH release in a dose-dependent manner. Meanwhile, the local and in vivo inhibition of hypothalamic neural nitric oxide synthase (nNOS) activity led to disruption in the rat oestrus cyclicity. In addition to long-term regulation by the hypothalamic-pituitary axis, autonomic innervation represents a form of rapid and direct regulation of ovarian function that can be an important adaptation of female reproductive function to internal or external environmental changes [4]. In rats, the main source of noradrenaline (NA) in the ovary is the superior ovarian nerve (SON); its fibers originate from the coeliac ganglion (CG) and are associated with follicular development and steroid secretion from the ovary [5, 6]. Recent findings support the view that these ganglia are complex integrative centers that receive inputs from central and peripheral sources with an abundance of neurochemical mediators, including NO [7]. Specifically, the nNOS enzyme has been detected in fibers that innervate the CG [7, 8], and it has been reported that NO plays a role as a neurotransmitter and neuromodulator of synaptic transmission [9, 10]. In adition, the presence of the GnRH receptor was observed in coeliac and superior mesenteric ganglia of goats [11]. Our group has already stardardized an integrated ex vivo system composed of the coeliac ganglion-superior ovarian nerve-ovary (CG-SON-Ovary), which mimics well the in vivo condition. This allows studying the interrelation of neural and endocrine phenomena on the physiology of the ovary. Using this model system, we reported that the CG is able to respond to different stimuli that modify the ovarian physiology through the SON in different reproductive states of the rat [10, 12–15]. Recently, we demonstrated the presence of GnRH/GnRH-receptor system in the CG and provided evidence for the fact that GnRH from the CG may trigger neuronal signals that promote luteal regression in late pregnant rats [16]. However, to the best of our knowledge, there are no references about the relatioship between NO and GnRH affecting the function of the sympathetic ganglia. Herein, an ex-vivo CG-SON-Ovary model obtained from rats on day 21 of pregnancy was used to test whether the inhibition of NO synthesis in the CG compartment affects the release of GnRH from the CG, thus modulating the physiology of the ovary at the end of pregnancy.

## Materials and methods

### Animals

Virgin Holtzman strain female rats weighing 250 ± 50 g were used on day 21 of pregnancy. They were kept in the bioterium of the National University of San Luis (San Luis, Argentina) under rigorous light conditions (12 h light, 07:00–19:00, and 12 h darkness), controlled temperature (22 ± 2 °C), with water and food ad libitum. The procedure used to induce gestation was described previously in detail [17]. Animals were handled according to the procedures approved in the UFAW Handbook on the Care and Management of Laboratory Animals. The experimental protocol was approved by the Institutional Animal Care and Use Committee of the National University of San Luis (protocol number B-264/17).

### Surgical and experimental procedures

**Fig. 1:**
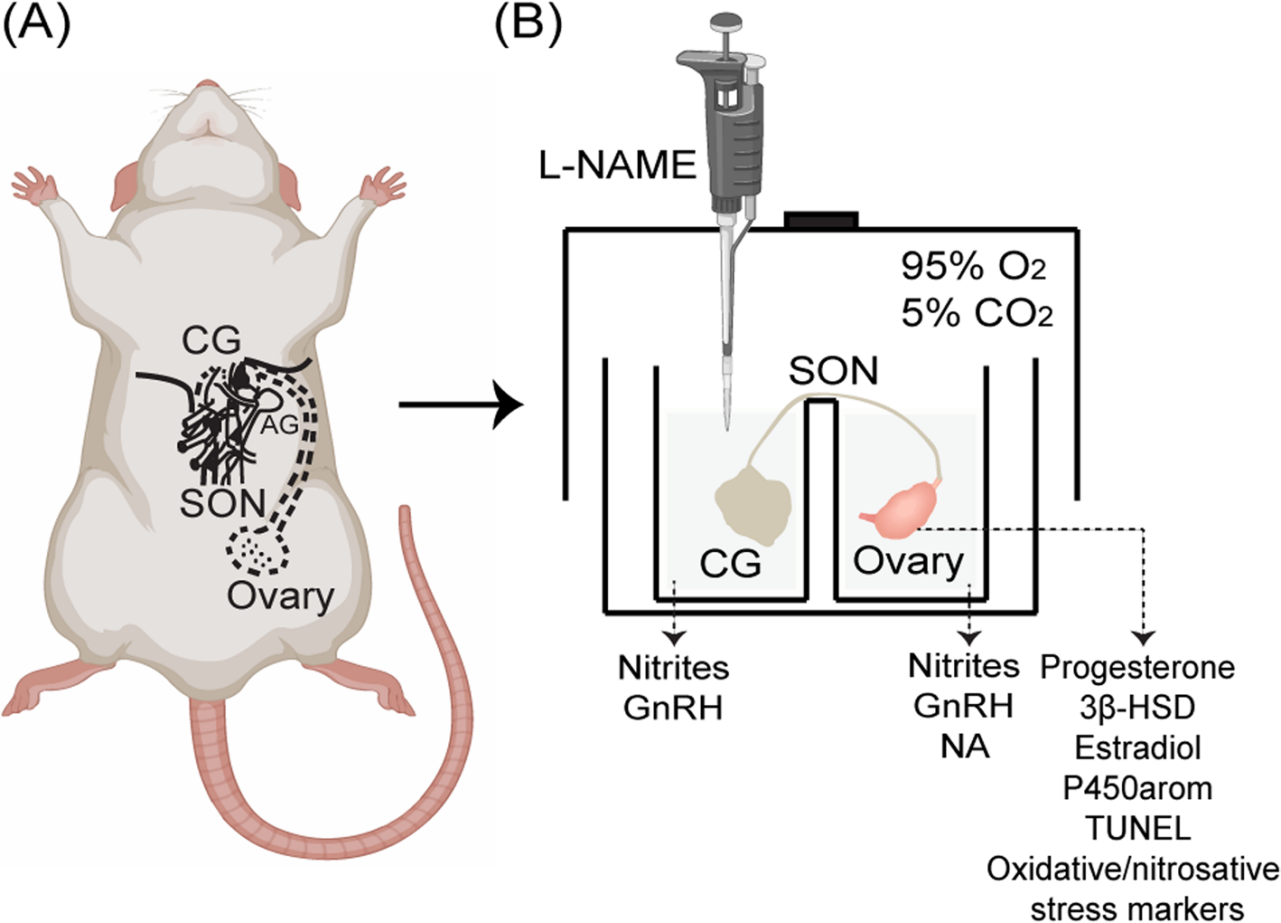
Schematic diagram of experimental model. **A** Schematic representation of the neural pathway of the superior ovarian nerve (SON) and the anatomical relationship with the coeliac ganglion (CG) and the ovary. The dashed lines indicate sites where the CG-SON-Ovary system was dissected out. **B** Cuvette used for the incubation of the ex vivo CG-SON-Ovary system with addition of L-NAME (100 μM) in the ganglionic compartment and main parameters analyzed. AG, adrenal gland; L-NAME, L-NG-nitro arginine methyl ester; NA, noradrenaline; 3β-HSD, 3β-hydroxysteroid dehydrogenase; P450arom, P450 aromatase

The surgical procedure to remove the CG–SON–Ovary system and the incubation conditions were conducted according to Casais et al. [17]. The animals were anaesthetized with 80 mg/kg of ketamine (Holliday Scott, Buenos Aires, Argentina) and 10 mg/kg of xylazine (Richmond, Buenos Aires, Argentina), intraperitoneally injected. The fetuses were removed and killed in an atmosphere of carbon dioxide. The CG–SON–Ovary system was extracted, and the mothers were sacrificed by decapitation. The entire CG–SON–Ovary system was removed by surgery, avoiding contact between the surgical instruments and the nerve fibers in order to prevent spontaneous nerve depolarization. The pieces of tissues removed consisted of the left ovary, the fibers that constituted the SON inserted in the suspensory ligament, and the CG accompanied by some small ganglia surrounding it. The entire surgical procedure was completed in 1–2 min. The CG–SON–Ovary system was placed in a cuvette with two isolated compartments, one for the CG and the other for the Ovary, both joined by the SON. The incubation medium used was Krebs-Ringer-bicarbonate buffer, with a pH of 7.4 with the addition of 0.1 mg/mL glucose and 0.1 mg/mL albumin at 37 °C, in an atmosphere composed of 95% O_2_ and 5% CO_2_. A schematic diagram of the experimental model was included in Fig.1. The ex vivo system was pre-incubated for 30 min, and the end of this period was considered incubation time 0. After this time, the buffer was replaced in both compartments, and 0.1 mg/mL ascorbic acid was added to the ganglion compartment as an antioxidant agent. The experimental group consisted in the addition in the ganglionic compartment of L-N^G^-nitro arginine methyl ester (L-NAME; Sigma-Aldrich, St. Louis, MO, USA), a non-selective NOS inhibitor, which was dissolved in 1 ml of Krebs-Ringer buffer at a final concentration of 100 μM [18]. The control group consisted of CG–SON–Ovary systems that were untreated. The incubation was performed for 180 min. At the end of this period, the ganglionic and ovarian incubation liquids were extracted and kept at − 20 °C until the measurement of NO and GnRH. Also, NA, progesterone, and estradiol were measured in the ovarian incubation liquids. Whole ovaries were weighed, and the corpora lutea were separated and stored at − 80 °C until protein extraction. Whole ovaries were also fixed in Bouin liquid for further analysis by a terminal deoxynucleotidyl transferase dUTP nick-end labelling (TUNEL) assay.

### Nitrite assay

Levels of nitrites, water-soluble metabolites of NO, were measured spectrophotometrically in the ganglionic and ovarian incubation liquids. Samples were immediately mixed with Griess reagent (sulfanilamide with N-1-naphthyl-ethylendiamine–HCL). After a 10 min incubation period at room temperature, the optical readings at 540 nm were measured and the nmol of nitrite were determined using a standard curve. The assay sensitivity was < 2.5 nmol/mL. The intraassay coefficients of variation for all the assays were < 10.0%. The results were expressed as nmol of nitrite per milligram of ovarian tissue (nmol/mg ovary).

### Radioimmunoassay (RIA)

GnRH in ganglionic and ovarian incubation liquids was measured by RIA. Intra- and inter-assay coefficients of variation were 7.1 and 11.6%, respectively, and the standard curve maintained linearity up to 100 pg/tube of GnRH.

Progesterone and estradiol levels were measured in the ovarian incubation liquid using a RIA kit (Beckman Coulter and DIAsource, respectively, DiagnosMed SRL, Buenos Aires, Argentina) following the manufacturer’s instructions. The inter-and intra-assay coefficients of variation in all the assays were < 10.0%.

### Catecholamine assay

Levels of NA in ovarian incubation liquids were measured by high performance liquid chromatography (HPLC; Prominence, Shimadzu, Japan) with electrochemical detection (Coulochem III, ESA, MA, USA) at IACA Laboratorios (Buenos Aires, Argentina).

### Enzyme-linked immunosorbent assay (ELISA)

Protein extracts were obtained using radioimmunoprecipitation assay buffer (RIPA buffer). The total protein concentration in corpora lutea homogenates was measured by the Bradford method.

The luteal expression of 3β-hydroxysteroid dehydrogenase (3β-HSD), an enzyme involved in progesterone synthesis, P450 aromatase (P450arom), involved in estradiol synthesis, and nitrotyrosine, a biomarker for the damage induced by NO-derived reactive nitrogen intermediates, were all analysed by enzyme-linked immunosorbent assay (ELISA). Aliquots containing 30 μg of total proteins for 3β-HSD, 20 μg of total proteins for P450arom, and 15 μg of total proteins for nitrotyrosine, were mixed with 0.1 M bicarbonate buffer with a pH of 9.6, in clear 96-well microplates (Corning Incorporated, Corning, NY, USA) and incubated for 1 h at 37 °C. After washing the sample with 0.05% (v/v) Tween 20 in phosphate buffered saline (PBS), and blocking it with 5% (w/v) non-fat dry milk in PBS with 0.05% (v/v) Tween 20 for 1 h at 37 °C, the microplates were incubated overnight at 4 °C with 100 μL of goat polyclonal anti-3β-HSD, 100 μL of rabbit polyclonal anti-P450arom (1:500; sc-30,820 and sc-30,086, Santa Cruz Biotechnology Inc., Santa Cruz, CA, USA), and 100 μL of rabbit polyclonal anti-nitrotyrosine antibody (1:1000; Sigma, St. Louis, MO, USA), respectively. After three washes, 100 μL of donkey anti-goat IgG peroxidase-linked antibody (1:500; sc-2020, Santa Cruz Biotechnology Inc., Santa Cruz, CA, USA), 100 μL of goat anti-rabbit IgG–horse-radish peroxidase (HRP) conjugate (1:5000; sc-2004, Santa Cruz Biotechnology Inc., Santa Cruz, CA, USA), and 50 μL of goat anti-rabbit IgG-HRP conjugate (1:4000 dilution; Jackson Immuno Research Laboratories, West Grove, PA, USA) were added to each well and incubated for 1 h at 37 °C. Finally, immunocomplexes were quantified using 3,3′,5,5′-Tetramethylbenzidine (TMB). The oxidation reaction of the substrate was terminated with 2 M sulfuric acid, and the optical density at 450 nm was measured using a TECAN microplate reader (Infinite M200 PRO, Research Triangle Park, NC, USA). The results were expressed in arbitrary units.

### TUNEL assay

The ovaries were processed for conventional optical microscopy; fixed in Bouin liquid, dehydrated in ethyl alcohol of increasing concentration, rinsed in xylol and included in paraffin. Four micrometer thick sections were generated with a Microm HM325 rotation microtome. Histological ovarian sections were processed for in situ localization of nuclei that exhibited DNA fragmentation using the In Situ Cell Death Detection Kit POD TUNEL assay (Cat N° 11,684,817,910 Roche, Basel, Switzerland), according to the manufacturer’s instructions. Apoptotic cells were visualized as immunolabelled intense brown structures after reaction with chromogen 3–3 ‘diaminobenzidine (DAB). Negative controls included omission of TdT. The positive control sections were incubated with 10 IU/mL DNase II (Sigma-Aldrich, St. Louis, MO, USA) in 50 mM Tris-HCL pH 7.5, 10 mM Mg_2_Cl, and 1 mg/mL BSA for 10 min at room temperature. Finally, the sections were counterstained with hematoxylin. The morphometric analysis of the histological sections processed with the TUNEL technique was performed using an Olympus BX40 optical microscope. Images were captured with a Sony SSC-DC5OA color camera and processed using the Image-Pro Plus 5.0 software. Three regularly spaced serial tissue sections (100 μm each) of corpora lutea corresponding to control and L-NAME groups were used and microscopic fields were examined under a 40X objective. In each section, 20 microscopic fields were randomly selected for each CL. The percentage of labeled cells was determined using the formula A/ (A + B) × 100, where A is the number of immunoreactive cells and B is the total number of unlabeled nuclei in the image. At least 1-hundred cells by field were counted by two independent observers, blinded to the experimental conditions. The results were expressed as a percentage of TUNEL positive cells, which was calculated per rat. All percentages were used to obtain the mean value per group.

### Measurement of lipid peroxidation

The thiobarbituric acid reactive substances (TBARS) assay measures malondialdehyde (MDA) production from lipid hydroperoxides. A calibration curve was performed using 1,1,3,3-tetramethoxypropane as standard. TBARS were determined by the absorbance at 535 nm and were expressed as μmol of MDA per milligram of total proteins (μmol MDA/mg protein).

### Total antioxidant capacity

Total antioxidant capacity (TAC) was measured by an improved method of bleaching of the 2,2′-azino-bis-(3-ethylbenzothiazoline-6-sulfonic acid) radical cation (ABTS^●+^) by both lipophilic and hydrophilic antioxidants present in the protein extracts. The ABTS^●+^ was generated by oxidation of 7 mM ABTS with 2.45 mM potassium persulfate. The TAC was expressed as the percentage of reduction in the absorbance due to the ABTS^●+^, and it was determined as follows: % inhibition = [(A0-Af)/ A0] × 100, where A0 and Af are the absorbances at 734 nm of the reaction mixtures measured at t = 0 and after 5 min of sample addition, respectively. All measurements were performed in duplicate for each sample.

### Statistical analysis

Statistical analysis was performed using GraphPad Prism (Version 5, GraphPad Software Inc. San Diego, CA, USA). All data are presented as the mean values ± standard error of the mean (S.E.M) in each group. Differences between two groups were analysed with Student’s unpaired *t* test. To analyze the percentage of positive TUNEL, a non-parametric (Mann–Whitney) test was used. A value of *p* < 0.05 was considered statistically significant. When comparing more than two groups, we used either one-way ANOVA followed by the Tukey’s multiple comparison test, or two-way ANOVA followed by the Bonferroni’s multiple comparison test.

## Results

### Nitric oxide and GnRH ganglionic levels

**Fig. 2:**
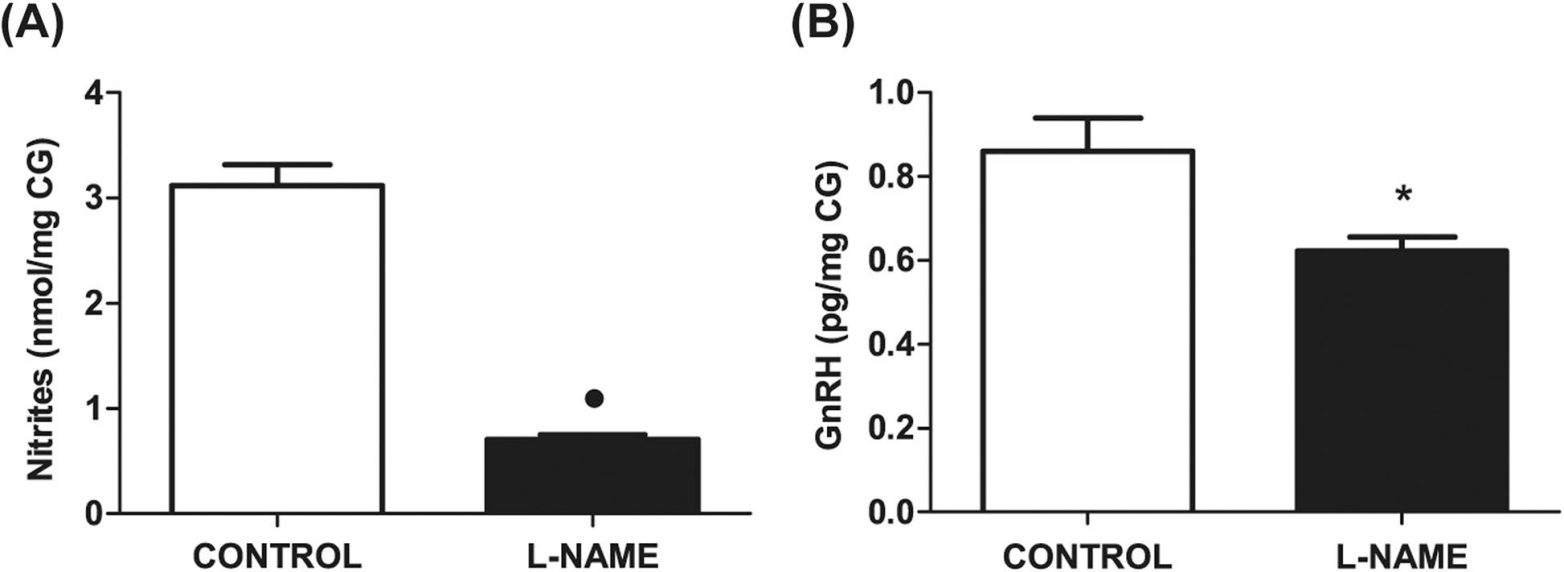
Ganglionic effect of L-NAME on NO and GnRH ganglionic release. **A** Ganglionic nitrites release, expressed in nmol/mg CG. **B** Ganglionic GnRH release, expressed in pg/mg CG. The values represent the mean ± S.E.M. of 6 animals per experimental group. Student’s unpaired *t* test: (●) *P* < 0.01; (*) *P* < 0.05

In order to determine a possible role for NO in the release of GnRH in the CG, we first confirmed the NO release in the ganglionic compartment of CG-SON-Ovary organ complexes of control pregnant rats. Then, the addition of the NO synthesis blocker L-NAME to the ganglionic incubation medium effectively decreased the levels of nitrites (*p* < 0.01) (Fig. 2A), as well as GnRH (*p* < 0.05), compared to the control group (Fig. 2B), which suggests that NO may play a role in the release of GnRH in the CG.

### Nitric oxide, GnRH and NA ovarian levels

**Fig. 3:**
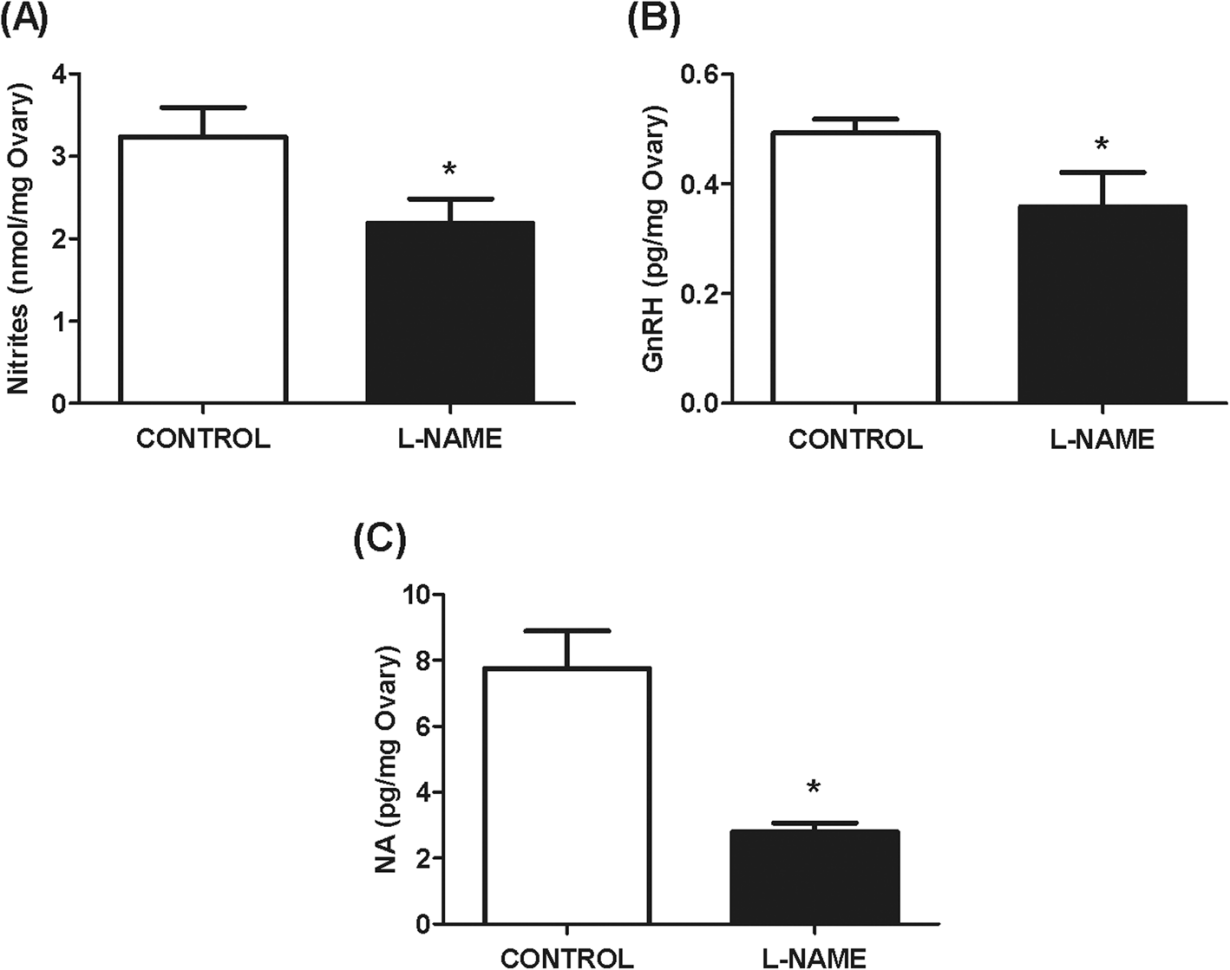
Ganglionic effect of L-NAME on nitrites, GnRH, and NA ovarian release. **A** Ovarian nitrites release, expressed in nmol/mg ovary. **B** Ovarian GnRH release, expressed in pg/mg ovary. **C** Ovarian noradrenaline (NA) release. The results were expressed as pg/mg ovary. The values represent the mean ± S.E.M. of 6 animals per experimental group. Student’s unpaired *t* test: (*) *p* < 0.05

NO, GnRH, and NA are neurotransmitters that participate in the modulation of ovarian physiology. The presence of L-NAME in the ganglion compartment significantly decreased the ovarian release of these neurotransmitters with respect to the control group (*p* < 0.05) (Fig. 3A-C).

### Ovarian levels of progesterone and luteal expression of 3β-HSD

**Fig. 4:**
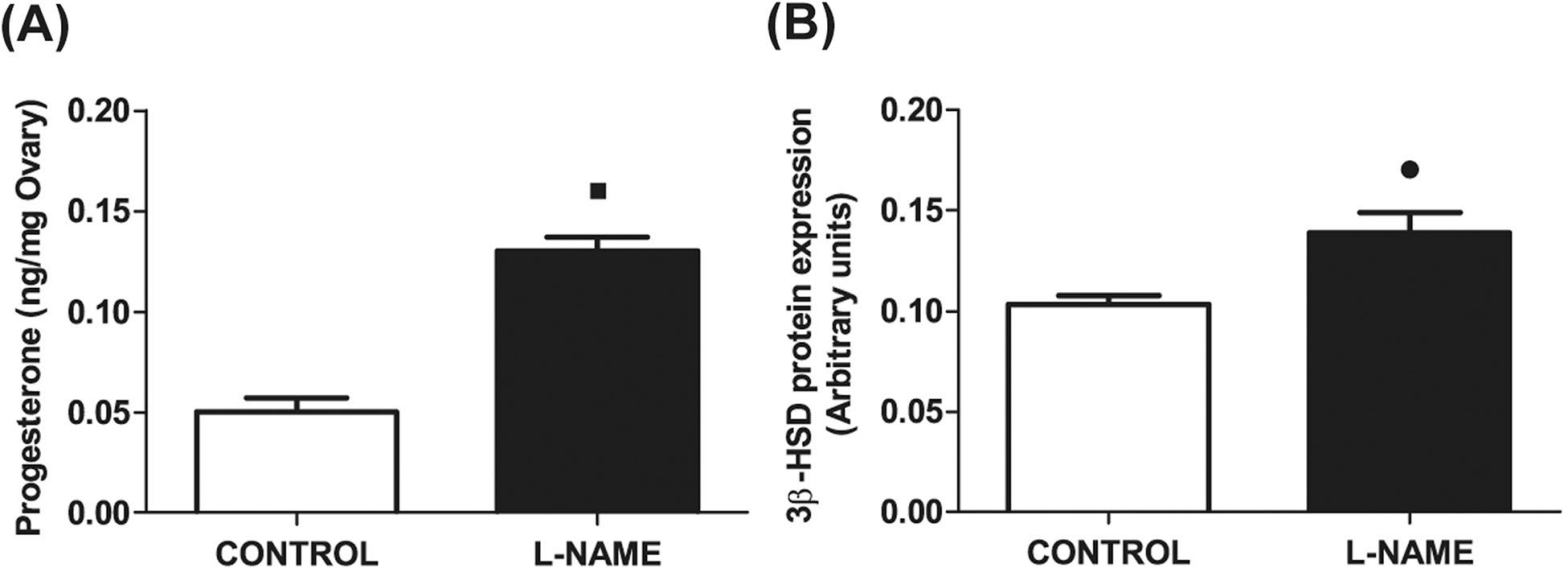
Ganglionic effect of L-NAME on progesterone ovarian levels and luteal expression of 3β-HSD. **A** Ovarian progesterone release, expressed in ng/mg ovary. **B** Protein expression of the progesterone synthesis enzyme, 3β-HSD, expressed in arbitrary units. The progesterone release values represent the mean ± S.E.M. of 6 animals per experimental group and 3 animals per experimental group for 3β-HSD expression. Student’s unpaired *t* test: (∎) *P* < 0.001; (●) *P* < 0.01

Given the previous findings by our group concerning the luteolytic effect of GnRH released from the CG on the pregnant rat ovary, we analysed the levels of the main luteal ovarian steroid—progesterone—and the luteal expression of its biosynthetic enzyme (3β-HSD). We observed that blocking NO synthesis in the CG compartment caused an increased release of progesterone in the ovarian compartment (*p* < 0.001) (Fig. 4A), while increased the expression of luteal 3β-HSD (*p* < 0.01) (Fig. 4B).

### Ovarian levels of estradiol and luteal expression of P450arom

**Fig. 5:**
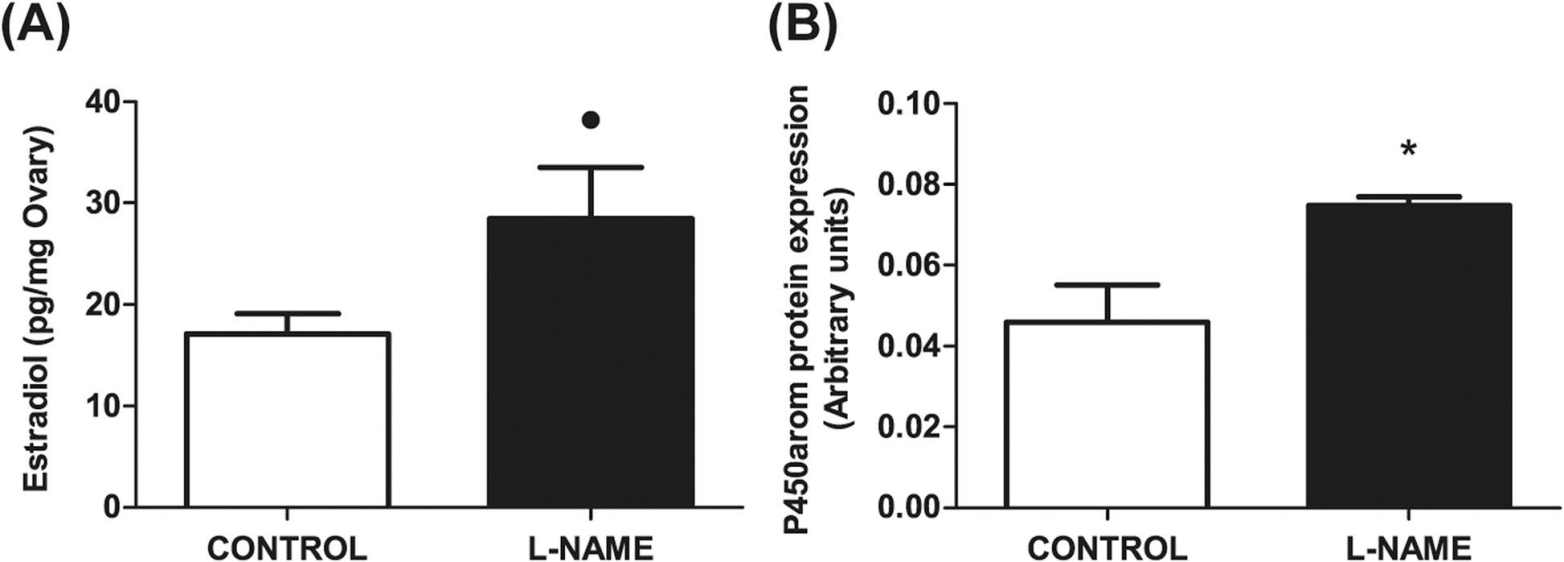
Ganglionic effect of L-NAME on ovarian levels of estradiol and luteal expression of P450arom. **A** Ovarian estradiol release, expressed in pg/mg ovary. **B** Protein expression of estradiol synthesis enzyme, P450arom, expressed in arbitrary units. The values represent the mean ± S.E.M. of 6 animals per experimental group for estradiol release and 3 animals per experimental group for P450arom expression. Student’s unpaired *t* test: (●) *P* < 0.01; (*) *P* < 0.05

Estradiol is another key hormone regulating reproductive processes when acting on the CL. The addition of L-NAME in the ganglionic compartment increased the estradiol released in the ovarian compartment (*P* < 0.01) (Fig. 5A), as well as the expression of its limiting biosynthetic enzyme, P450arom, when compared to the control group (*P* < 0.05) (Fig. 5B).

### Luteal apoptosis

**Fig. 6:**
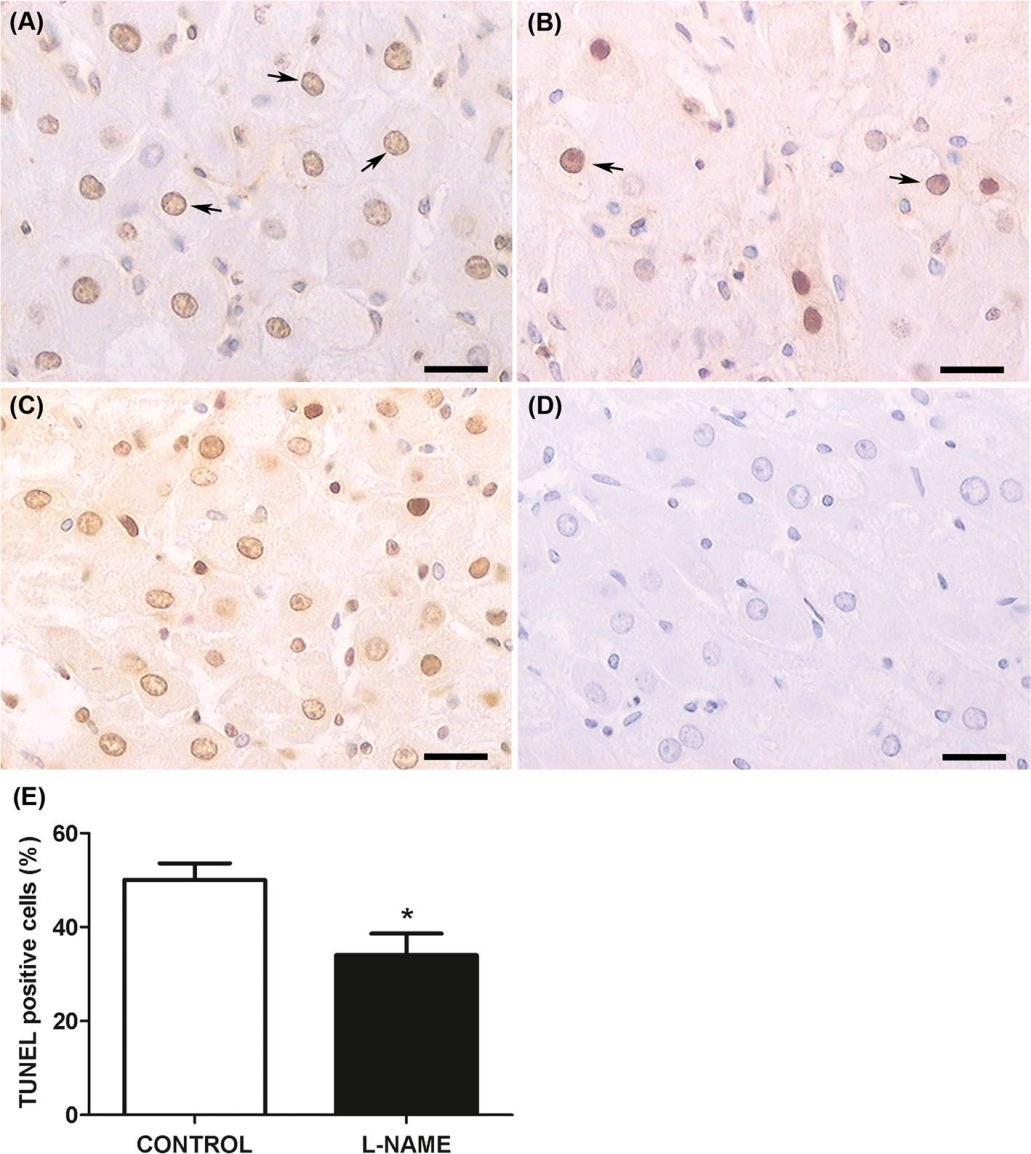
Ganglionic effect of L-NAME on luteal apoptosis assessed by TUNEL. Representative micrographs of CL histological sections corresponding to control group (**A**), L-NAME group (**B**), positive control of the technique (**C**), and negative control (**D**). Arrows indicate positive TUNEL brown apoptotic nuclei. Contrast coloration: Hematoxylin, total magnification X400. Bar scale: 25 μm. **E** Apoptosis levels expressed as percentage of TUNEL positive cells. Values represent the mean ± S.E.M of 4 animals per experimental group. Mann-Whitney test: (*) *P* < 0.05

After analyzing luteal steroidogenesis markers, we evaluated apoptosis as a marker of luteal structural regression. The corpora lutea isolated from the CG-SON-Ovary systems incubated with the addition of L-NAME in the ganglionic compartment showed a lower percentage of apoptotic cells in the ovarian compartment than the control group (*p* < 0.05) (Fig. 6).

### Oxidative status

**Fig. 7:**
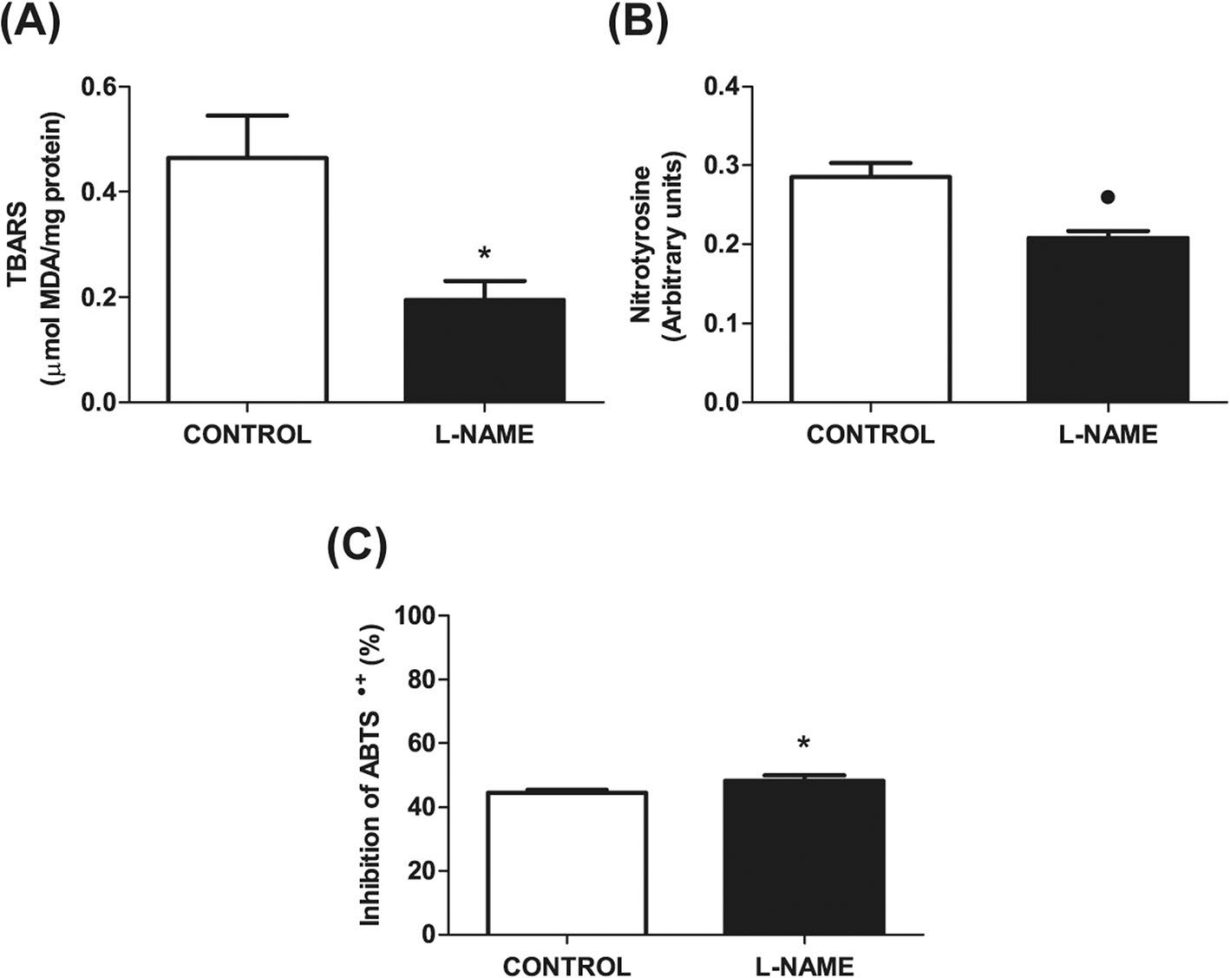
Ganglionic effect of L-NAME on luteal markers of oxidative/nitrosative damage and total antioxidant capacity. **A** Thiobarbituric acid reactive substances (TBARS) concentration, expressed as μmol MDA/mg protein. **B** Nitrotyrosine content, expressed in arbitrary units. **C** Total antioxidant capacity (TAC), measured as percentage of inhibition of the ABTS●+. The values represent the mean ± S.E.M. of 6 animals per experimental group. Student’s unpaired *t* test: (●) *P* < 0.01; (*) *P* < 0.05

We further analysed oxidative/nitrosative stress markers in the CL. The addition of L-NAME into the CG compartment significantly decreased the levels of TBARS (*p* < 0.05) (Fig. 7A), as well as nitrotyrosine (*p* < 0.01) (Fig. 7B). In addition, there was a significant increase in the TAC when compared to the control group (*p* < 0.05) (Fig. 7C).

## Discussion

GnRH and NO are signaling mediators that participate in the regulation of reproductive functions. Herein, by using an ex-vivo CG-SON-Ovary system we studied whether blocking NO synthesis in the CG affects the release of GnRH from the CG, and the impact on the physiology of the ovary at the end of pregnancy. Previous results from our research group demonstrated that the addition of the GnRH antagonist cetrorelix (CTX) to the ganglionic compartment of the CG-SON-Ovary system increased the ganglionic GnRH levels, indicating the presence of a functional GnRH system in the CG [16]. Surprisingly, in that study we also found an increase in the levels of NO in the ganglionic compartment. Such increase could be a compensatory mechanism due to the blockage of the GnRH receptors and suggested a potential relationship between NO and GnRH release in the sympathetic ganglia.

Previous studies have suggested that NO is a key modulator of GnRH secretion from the hypothalamus, a necessary process for normal ovarian function and reproductive cyclicity [19, 20]. In sympathetic ganglia, the presence of the NO/NOS system has been established [7, 8]. Furthermore, the use of drugs that block NO synthesis, such as L-NAME, have shown that endogenously released NO can modulate synaptic transmission [9]. In the present study, the addition of L-NAME decreased NO and GnRH levels in the ganglionic compartment. Therefore, these data suggest that NO affects the release of GnRH in the CG. Furthermore, we studied whether the joint blockage of the action of GnRH and the production of NO in the CG, through the combined addition of L-NAME and CTX in the ganglionic compartment, modifies the accumulation of GnRH. The results obtained indicate that the joint addition of L-NAME and CTX in the CG decreased the levels of GnRH in the ganglion (Supplementary Fig. 1A) and ovarian (Supplementary Fig. 1B) compartments compared to the control group. A comparative statistical analysis between the different experimental groups indicates the prevalence of the effect of L-NAME over CTX, reinforcing the role that NO plays in the accumulation of GnRH in the ganglionic compartment.

The inhibition of NO production in the CG compartment with L-NAME decreased the release of the gaseous neutrotransmitter in the ovarian compartment. This agrees with results showing a time-dependent decrease in nitrites levels upon 100 μM L-NAME treatment using the same multiorgan system in cycling rats (Supplementary Fig. 2). In addition to blocking the accumulation of GnRH in the ganglionic compartment, the addition of L-NAME in the CG compartment led to a decreased release of GnRH in the ovarian compartment. Taken together, these data suggest a link between NO and GnRH at the ganglionic and ovarian levels and, given the presence of GnRH and NO systems in the ovary, an intraovarian regulatory mechanism should not be ruled out.

Several studies have shown that GnRH may be an important regulator of the regression of the CL. Thus, the administration of GnRH agonists inhibits progesterone production and induces apoptosis in the CL of pregnant rats; such anti-steroidogenic effect may be due to the inhibitory effect on the enzymes involved in the steroidogenic pathway [21, 22]. In addition, an in vitro study showed a suppressive effect of a GnRH agonist on ovarian progesterone synthesis associated with decreased 3β-HSD in the ovary of mice [23]. In agreement with this study, we demostrated that the increased progesterone biosynthesis in the CL was accompanied by an increase in the luteal expression of its limiting biosynthesis enzyme, 3β-HSD.

NA is a neurotransmitter that regulates ovarian steroidogenesis. Stimulatory or inhibitory effects of NA on steroid hormone secretion were reported, depending on what type of adrenoreceptors is activated in the ovary [24]. The decrease in the release of NA from the ovary upon blocking NO synthesis in the CG indicates that, in addition to GnRH, NA may be one of the transmitters involved in the increase of progesterone production. In agreement with our results, Ramírez Hernández et al. [25] showed that sectioning of the SON reduces the levels of NA in the ovary, and that this effect is accompanied by an increase in the biosynthesis of progesterone. Regarding estradiol, Sridaran and Mahesh [26] demonstrated that GnRH has no effect on its luteal synthesis. However, it was demonstrated that the synthesis of estradiol is regulated negatively by NO in cells obtained from luteinized ovaries [27]. Therefore, the decrease in ovarian NO production by L-NAME in the CG compartment may be responsible for the increase in estradiol biosynthesis and P450arom expression in the CL. These results were accompanied by a decrease in GnRH levels. This neuropeptide has not been detected in ovarian nerve endings so far. However, the presence of a GnRH system in the ovary is known, which is negatively regulated by estradiol [15, 28]. Therefore, in the present study, NO may regulate ovarian GnRH release through estradiol.

Apoptosis is one of the main mechanisms of celular death involved in the regression of the CL [2, 29]. Some reports indicate that the structural luteal regression begins before the functional luteal regression is complete [30, 31]. In addition, a modest number of apoptotic cells are present during the late luteal stage, which increases in the early postpartum period [32, 33]. Several studies support the protective role of progesterone in the function and survival of the CL, since it can locally stimulate its own production to protect the CL from cell death [2, 34, 35]. By contrast, GnRH may facilitate apoptosis and CL regression [16, 36, 37]. The reduced apoptotic levels found in the CL in the current study are consistent with an increase in the release of progesterone and a decrease in the release of GnRH into the medium of ovarian incubation of L-NAME-treated systems in the CG. In turn, the release of NA decreased in this experimental group. In addition, there is evidence that NA is involved in the regulation of ovarian apoptosis [15, 38]. Previous results from our laboratory indicate that on diestrus II, a stage in which the CL undergoes regression, the addition of NA in the ovarian incubation medium regulates ovarian steroidogenesis by modulating the release of GnRH, thus favoring apoptosis [15].

During luteal regression, the generation of ROS, which may be attributed to both accelerated generation and inefficient removal, has been associated with decreased progesterone production and induction of cell death [39–41]. In the present study, a decrease in the lipid peroxidation products and the nitrotyrosine concentration, together with low levels of NO, was observed in ovaries from systems incubated with L-NAME in the CG compartment. Regarding NO, both luteotrophic and luteolytic effects have been attributed to this molecule [42–44]. Furthermore, it has been suggested that the effect of NO on cellular processes depends on its concentration and the presence of other free radicals. Thus, the proapoptotic effect of NO is mainly associated with high concentrations [45, 46]. Therefore, the low levels of ovarian NO would contribute to the low percentage of apoptotic cells seen in the L-NAME group. Nitrotyrosine is indicative of formation and activity of the NO-derived oxidant peroxynitrite; therefore, it serves as a marker of NO metabolism [47]; it should be noted that its concentration also decreased in this experimental group.

Regarding lipid peroxidation, several studies indicate that its increase inhibits progesterone production in luteal cells and is a trigger for luteal regression [39, 48]. In the present study, in addition to the decrease in the levels of these oxidative/nitrosative damage markers, an increase in the TAC was found in the corpora lutea isolated from the CG-SON-Ovary systems incubated with L-NAME in the CG compartment. The ovarian tissue contains enzymatic and non-enzymatic antioxidant systems, responsible for eliminating the excess of ROS, which could be the subject of endocrine regulation [49, 50]. It should be noted that the aforementioned results occurred with an increase in estradiol levels, to which several studies have attributed antioxidant properties in the luteal cells [51, 52]. For example, Vega et al. [53] reported that the addition of estradiol to human luteal cells incubated in vitro led to a decrease in TBARS levels.

It is not yet well established which factors have the greatest contribution to the inhibition of progesterone synthesis, activation of the signaling pathway of apoptosis, and regression of the CL. However, it was suggested by other authors that the end of luteal functionality depends on the balance among several factors [1, 48]. In a previous study, we provided evidence that GnRH released from in the CG might trigger neuronal signals that promote luteal regression in late pregnancy [16]. In the present study, we showed that NO affects GnRH release from the CG and the consequent impacts on the physiology of the ovary. Together, these findings highlight the functional role of these factors through the peripheral innervation in the regulation of ovarian function. Given that the modulation of the NO system can be a useful tool in the process of induction of labor, in addition to the known effect on the uterus, the contribution of the peripheral neural pathway and its influence on ovarian steroid hormone production should be taken into account.

## Conclusions

The inhibition of NO production by L-NAME in the GC lead to a reduction in ganglionic GnRH release and caused changes that promoted luteal wellbeing, associated with a decrease in ovarian levels of NO, GnRH and NA, possible neurotransmitters involved in the observed effects. These observations suggest that the production of NO by the CG modulates the physiology of the ovary and the regression of the CL during late pregnancy in rats.

## Supplementary Information

**Additional file 1: Supplementary Figure 1.** Effect of the combined addition of L-NAME (100 μM) and CTX (10^− 6^ M) in CG on the release of GnRH, at 180 min. Incubation of the CG-SON-Ovary system of rats with 21 days of pregnancy. A) Ganglionic GnRH release, expressed in pg/mg CG. B) Ovarian GnRH release, expressed in pg/mg Ovary. The Control group consisted of untreated CG-SON-Ovary systems. Values represent the mean ± S.E.M. of 6 animals per experimental group. One-way ANOVA followed by the Tukey’s multiple comparison tests: (*) *P* < 0.05; (●) *P* < 0.01; (■) *P* < 0.001.

**Additional file 2: Supplementary Figure 2.** Effect of stimulation of the CG with 100 μM L-NAME on the concentration of nitrites in the incubation medium of the left ovary, during the first proestrus in the rat. For this, the CG-SON-Ovary system was extracted from 37-day-old Holtzman rats and immediately placed in a cuvette with two compartments, one for the CG and the other for the ovary, both joined by the SON. The incubation medium was 1 ml of Krebs-Ringer bicarbonate buffer, pH 7.4, with 0.1 mg/ml dextrose and 0.1 mg/ml BSA at 37 °C in a saturated atmosphere of 95% O_2_ and 5% CO_2_. The results show that the addition of this nitric oxide synthase inhibitor in the ganglion compartment decreases the nitrite concentration in the ovarian incubation medium, throughout 180 min of incubation. The values represent the mean ± SEM of 6 animals per experimental group. Two-way ANOVA followed by Bonferroni’s multiple comparison tests: (*) *P* < 0.05; (■) *P* < 0.001.

## Data Availability

The datasets used and/or analysed during the current study are available from the corresponding author on reasonable request. This work is part of the CONICET Doctoral Scholarship and the Doctoral Thesis of Laura Morales.

## Abbreviations

NO: Nitric oxide
GnRH: Gonadotrophin-releasing hormone
CG-SON-Ovary: Coeliac ganglion-superior ovarian nerve-ovary
L-NAME: L-NG-nitro arginine methyl ester
CL: Corpus luteum
CTX: Cetrorelix
ROS: Reactive oxygen species
nNOS: Neural nitric oxide synthase
NA: Noradrenaline
RIA: Radioimmunoassay
ELISA: Enzyme-linked immunosorbent assay
3β-HSD: 3β-hydroxysteroid dehydrogenase
P450arom: P450 aromatase
TBARS: Thiobarbituric acid reactive substances
MDA: Malondialdehyde
TUNEL: Terminal deoxynucleotidyl transferase dUTP nick-end labelling
