# Supplementary figures and images for: Interplay between nitric oxide and gonadotrophin-releasing hormone in the neuromodulation of the corpus luteum during late pregnancy in the rat
Source: Reprod Biol Endocrinol. 2022 Jan 26;20:19. doi: 10.1186/s12958-022-00894-6 (PMC8793209; doi:10.1186/s12958-022-00894-6)

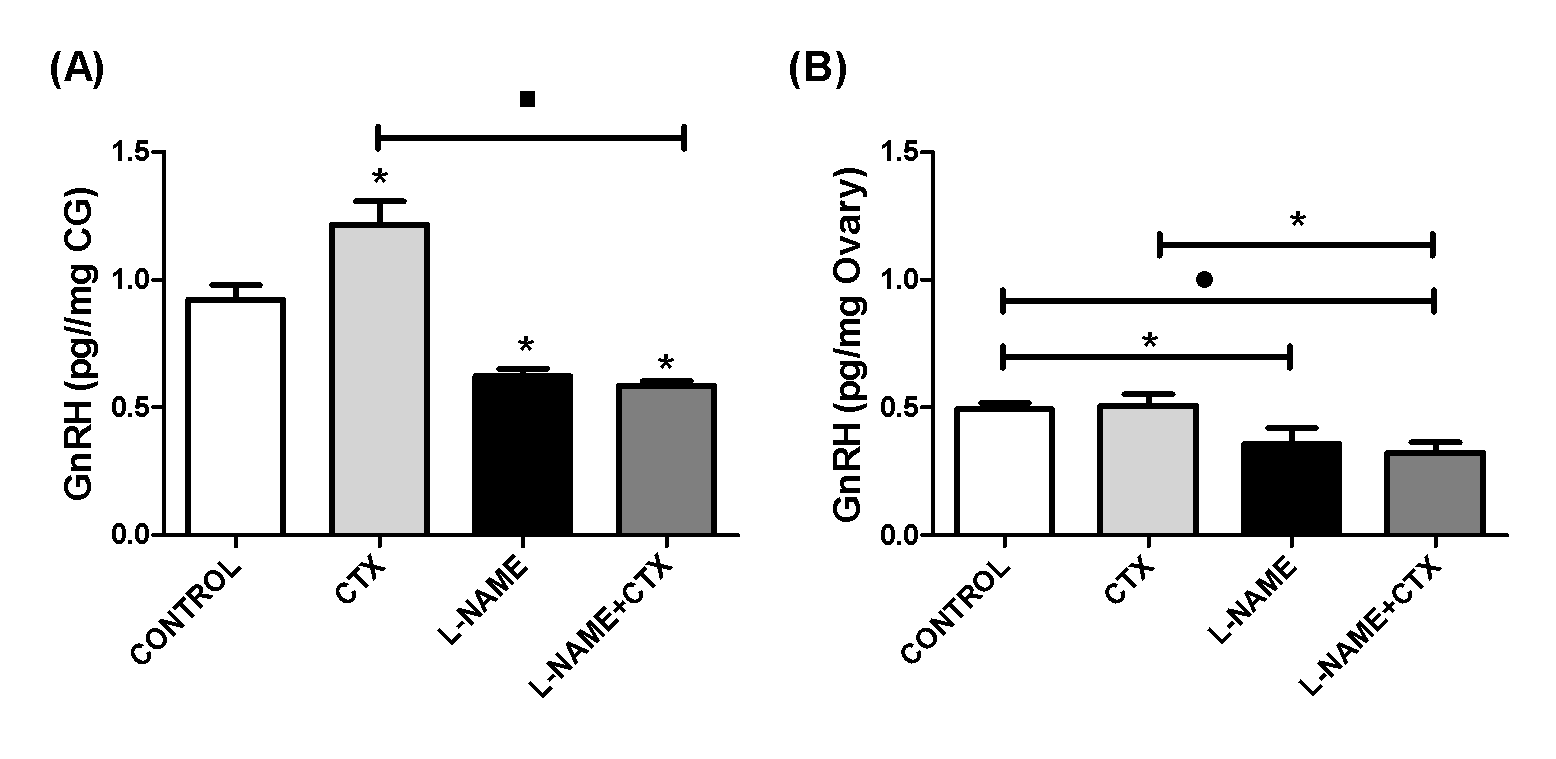

Supplement: Supplementary file 1 — Additional file 1: Supplementary Figure 1. Effect of the combined addition of L-NAME (100 μM) and CTX (10− 6 M) in CG on the release of GnRH, at 180 min. Incubation of the CG-SON-Ovary system of rats with 21 days of pregnancy. A) Ganglionic GnRH release, expressed in pg/mg CG. B) Ovarian GnRH release, expressed in pg/mg Ovary. The Control group consisted of untreated CG-SON-Ovary systems. Values represent the mean ± S.E.M. of 6 animals per experimental group. One-way ANOVA followed by the Tukey’s multiple comparison tests: (*) P < 0.05; (●) P < 0.01; (■) P < 0.001. [file 12958_2022_894_MOESM1_ESM.tif]

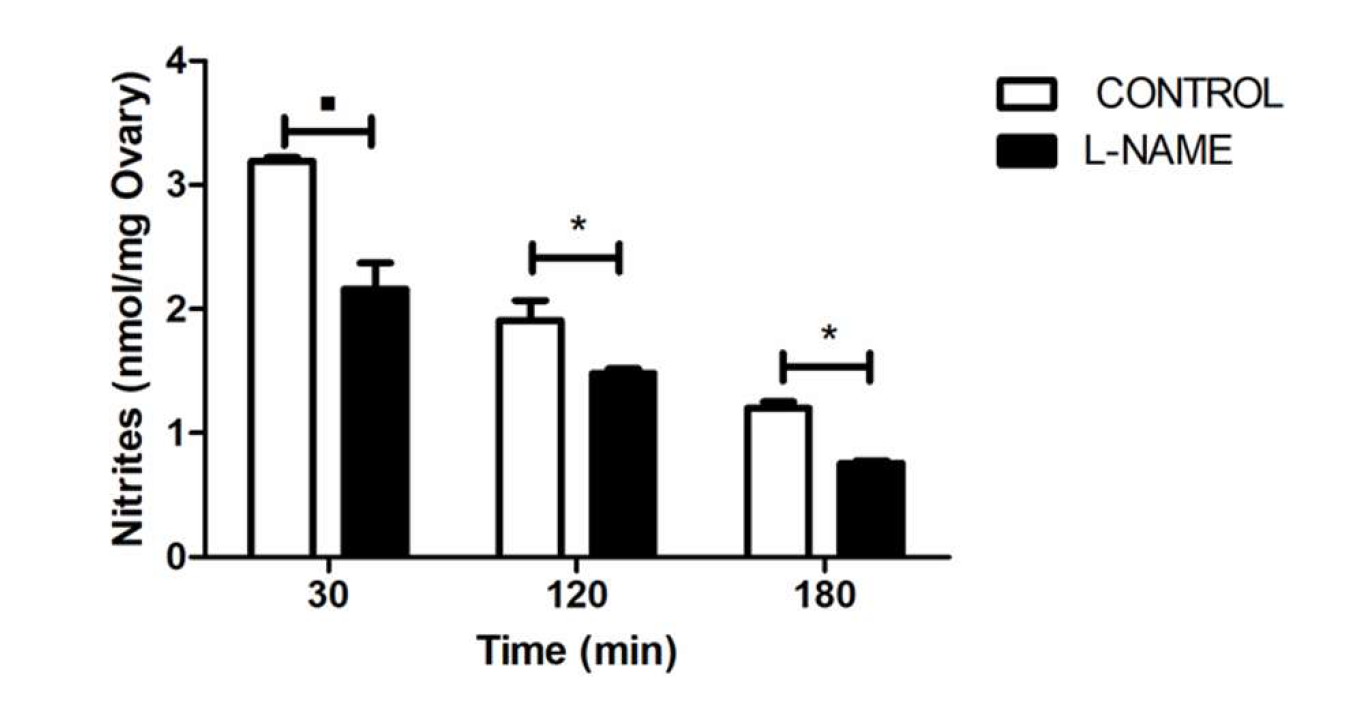

Supplement: Supplementary file 2 — Additional file 2: Supplementary Figure 2. Effect of stimulation of the CG with 100 μM L-NAME on the concentration of nitrites in the incubation medium of the left ovary, during the first proestrus in the rat. For this, the CG-SON-Ovary system was extracted from 37-day-old Holtzman rats and immediately placed in a cuvette with two compartments, one for the CG and the other for the ovary, both joined by the SON. The incubation medium was 1 ml of Krebs-Ringer bicarbonate buffer, pH 7.4, with 0.1 mg/ml dextrose and 0.1 mg/ml BSA at 37 °C in a saturated atmosphere of 95% O2 and 5% CO2. The results show that the addition of this nitric oxide synthase inhibitor in the ganglion compartment decreases the nitrite concentration in the ovarian incubation medium, throughout 180 min of incubation. The values represent the mean ± SEM of 6 animals per experimental group. Two-way ANOVA followed by Bonferroni’s multiple comparison tests: (*) P < 0.05; (■) P < 0.001. [file 12958_2022_894_MOESM2_ESM.tif]
